# Pantomime of tool use: looking beyond apraxia

**DOI:** 10.1093/braincomms/fcab263

**Published:** 2021-10-30

**Authors:** François Osiurak, Emanuelle Reynaud, Josselin Baumard, Yves Rossetti, Angela Bartolo, Mathieu Lesourd

**Affiliations:** 1 Laboratoire d’Etude des Mécanismes Cognitifs (EA3082), Université Lyon 2, 69676 Bron, France; 2 Institut Universitaire de France, 75231 Paris, France; 3 Normandie University, UNIROUEN, CRFDP (EA7475), 76821 Mont Saint Aignan, France; 4 Centre de Recherche en Neurosciences de Lyon, Trajectoires Team, CNRS U5292, Inserm U1028, Université de Lyon, 69676 Bron, France; 5 Mouvement, Handicap, et Neuro-Immersion, Hospices Civils de Lyon et Centre de Recherche en Neurosciences de Lyon, Hôpital Henry Gabrielle, 69230 Saint-Genis-Laval, France; 6 Univ. Lille, CNRS, UMR9193, SCALab—Sciences Cognitives et Sciences Affectives, 59653 Villeneuve d'Ascq, France; 7 Laboratoire de Recherches Intégratives en Neurosciences et Psychologie Cognitive (UR481), Université de Bourgogne Franche-Comté, 25030 Besançon, France; 8 MSHE Ledoux, CNRS, Université de Bourgogne Franche-Comté, 25000 Besançon, France

**Keywords:** pantomime, apraxia, tool use, technical reasoning, left inferior parietal lobe

## Abstract

Pantomime has a long tradition in clinical neuropsychology of apraxia. It has been much more used by researchers and clinicians to assess tool-use disorders than real tool use. Nevertheless, it remains incompletely understood and has given rise to controversies, such as the involvement of the left inferior parietal lobe or the nature of the underlying cognitive processes. The present article offers a comprehensive framework, with the aim of specifying the neural and cognitive bases of pantomime. To do so, we conducted a series of meta-analyses of brain-lesion, neuroimaging and behavioural studies about pantomime and other related tasks (i.e. real tool use, imitation of meaningless postures and semantic knowledge). The first key finding is that the area PF (Area PF complex) within the left inferior parietal lobe is crucially involved in both pantomime and real tool use as well as in the kinematics component of pantomime. The second key finding is the absence of a well-defined neural substrate for the posture component of pantomime (both grip errors and body-part-as-tool responses). The third key finding is the role played by the intraparietal sulcus in both pantomime and imitation of meaningless postures. The fourth key finding is that the left angular gyrus seems to be critical in the production of motor actions directed towards the body. The fifth key finding is that performance on pantomime is strongly correlated with the severity of semantic deficits. Taken together, these findings invite us to offer a neurocognitive model of pantomime, which provides an integrated alternative to the two hypotheses that dominate the field: The gesture-engram hypothesis and the communicative hypothesis. More specifically, this model assumes that technical reasoning (notably the left area PF), the motor-control system (notably the intraparietal sulcus), body structural description (notably the left angular gyrus), semantic knowledge (notably the polar temporal lobes) and potentially theory of mind (notably the middle prefrontal cortex) work in concert to produce pantomime. The original features of this model open new avenues for understanding the neurocognitive bases of pantomime, emphasizing that pantomime is a communicative task that nevertheless originates in specific tool-use (not motor-related) cognitive processes.

<Please insert Graphical abstract here>

## Teaser

Osiurak et al. describe neurocognitive bases of pantomime of tool use through a series of meta-analyses of brain-lesion, neuroimaging and behavioural studies.

## Graphical Abstract

**Graphical Abstract:**
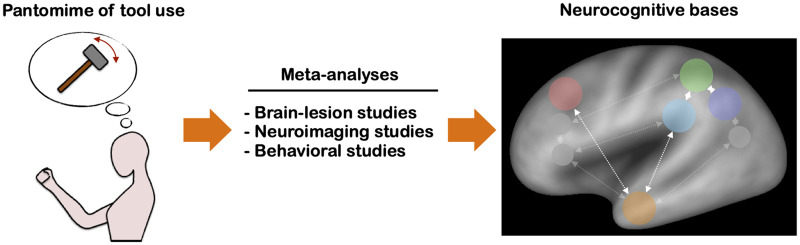

## Introduction

Limb apraxia—hereafter shortened as apraxia—refers to a high-level motor disorder that cannot be attributed to basic sensorimotor or comprehension deficits, and which disturbs the ability to imitate meaningless postures, produce symbolic gestures and/or use tools.^1–4^ Tool-use disorders can be investigated with real tool use (familiar or novel), single tool use and pantomime of tool use—hereafter shortened as pantomime (for a description of these tasks, see Table  1). Pantomime is a gold standard with a long tradition in clinical neuropsychology,^1^^,^^5–7^ which has been much more used by researchers and clinicians to assess tool-use disorders than real tool use.^8^^,^^9^ The main reason is that it can be viewed as a proxy for studying real tool-use disorders because the deficits are more salient than during real tool use.^10–19^ A secondary reason is its practical convenience. Surprisingly, even though pantomime has attracted considerable interest, it remains incompletely understood. Additionally, it has given rise to several controversies, such as the involvement of the left inferior parietal lobe (IPL) or the nature of the underlying cognitive processes. In this context, a comprehensive framework may be welcomed to shed some light on the rich and fascinating literature on pantomime.

**Table 1:** Description of tasks and hypotheses

Task	Description
Real tool use	The individual actually uses a tool^a^ (e.g. hammer) with an object ^a^ (e.g. nail). In some cases, several tools or objects are presented, and the individual must select the correct one to actually perform the tool-use action expected (i.e. tool selection).
Familiar tool use	Only familiar tools are presented. Familiar tool use differs from ‘activity of daily living’^70^ in which the individual has to perform a *sequence* of familiar tool-use actions. Note also that in familiar tool-use tasks, the individual has to use tool-object pairs. By contrast, activity-of-daily-living tasks involve multiple tools and objects, implying the selection of the tools and objects that are appropriate to perform the appropriate sequence of actions.
Novel tool use	The task can consist in using familiar tools in a non-conventional way (e.g. driving a screw with a knife) or in selecting, making and/or using novel tools to solve mechanical problems.
Single tool use	The individual grasps a tool presented in isolation and shows how to use it.
Pantomime of tool use	The individual demonstrates the use of a tool presented in isolation without holding it in hand.
Verbal modality	The name of the tool or a verbal description of the corresponding tool-use action is provided.
Visual modality	A picture of a tool or the real tool itself is shown.
Imitation modality	The examiner performs the tool-use action without holding the tool in hand.
Hypothesis	
Gesture engram	This hypothesis is grounded on the idea that all tool-use situations—including pantomime—critically require specific motor programs specifying the features of the movements to be performed to use a specific tool. These motor programmes have been labelled with different terms, such as visuo-kinesthetic motor engram,^20^ action lexicon,^214^ gesture engram^112^ or manipulation knowledge.^135^ We will hereafter use the generic term motor engram and, as a result, will call this perspective the gesture-engram hypothesis.
Communicative	This hypothesis highlights the important—if not exclusive—role of communicative/language skills in pantomime.
Mixed	This non-exclusive hypothesis suggests that both gesture engrams and communicative skills contribute to the production of pantomime. In other words, gesture engrams and communicative skills are complementary.
Technical reasoning	This hypothesis assumes that all tool-use situations—including pantomime—require reasoning about the physical properties of tools and objects to generate a mental simulation of the mechanical action appropriate to perform the task. Then, this mental simulation orients the selection, planning and online control of the appropriate motor actions within the motor-control system. This hypothesis does not exclude that additional cognitive processes can be at play in pantomime because of the absence of specific information.

a The term tool will be hereafter used for the implement that performs an action (e.g. hammer) and the term object for the recipient of the action (e.g. nail).

The present article aims at offering such a renewed framework, with the aim of specifying the neural and cognitive bases of pantomime. To do so, we conducted a series of meta-analyses of brain-lesion, neuroimaging and behavioural studies (see Supplementary Methods), which allowed us to test several hypotheses, and specifically the two dominant hypotheses in the field: The gesture-engram hypothesis and the communicative hypothesis (Table  1). The influential gesture-engram hypothesis assumes that gesture engrams are critical to any tool-use situations. In this frame, pantomime is the ideal task to investigate the integrity of motor engrams.^7^^,^^20–26^ By contrast, the communicative hypothesis questions the autonomy of apraxia from aphasia in highlighting the important—if not exclusive—role of communicative/language skills in pantomime.^8^^,^^12^^,^^27–29^ Our analyses provide conclusive support for a third alternative, namely that pantomime is a communicative task that nevertheless originates in specific tool-use (*not motor-related*) cognitive processes. This conclusion may appear trivial for some contributors, who have already stressed that pantomime is at the crossroad between tool use and communicative skills^30^ and that it should be more fruitful to consider the two hypotheses as complementary (i.e. the mixed hypothesis^31^). However, our conclusion offers a subtle but perhaps crucial nuance, in ruling out the gesture-engram component (hence the ‘*not motor-related*’ above) and in arguing for a rival hypothesis, namely, the technical-reasoning hypothesis^32^^,^^33^ (Table  1). In broad terms, the alternative hypothesis proposed here goes beyond the mixed hypothesis. To capture its essence, one may be instructed to temporarily discard the idea that pantomime is a clinical task aiming to assess the integrity of so-called motor engrams. We acknowledge that this statement is provocative, notably because of the strong dominance of the motor-engram hypothesis in the literature. In addition, challenging the concept of gesture engrams amounts to question the very concept of apraxia. After all, what may a high-level motor disorder become if we do not hypothesize the existence of a high-level motor component such as motor engrams? We will come back to this possibility later. For the sake of simplicity, we will temporarily keep this assumption aside to explore alternative ways of understanding the neurocognitive origins of pantomime. Keeping this in mind, let us begin by defining pantomime.

## Definition

Pantomime refers here to a mime consisting in pretending to use a tool as if it was held in hand (e.g. pretending to brush teeth with a toothbrush or to pound a nail with a hammer). Although pantomime can be considered as gesture, it must not be confounded with other expressive and communicative movements.^34^ Thus, pantomime does not concern conventionalized movements that are embedded in a situation, such as symbolic gestures (e.g. waving goodbye), nor does it concern a gesture accompanying speech.^35^^,^^36^ It must also not be confounded with signs that are used like words in connected discourse (e.g. sign language^37^).

## The function of pantomime

The question of the function of pantomime is important. Yet, it has received little attention to date, reflecting that it is mainly viewed as a clinical task. The motor-engram hypothesis has been developed based on the idea that pantomime is a proxy for studying real tool-use disorders. Thus, the error scoring system describes the errors committed on parameters that are transposable to real tool use^13^^,^^38–42^ (e.g. hand posture, arm trajectory, amplitude, timing). The motor-engram hypothesis does not elaborate on the function of pantomime, otherwise than as an ideal task to assess the integrity of motor engrams. Yet, we rarely pantomime in everyday life. Just take a few minutes to think about how many times you have produced a pantomime over the last 3 days. You may realize that this number is very low and that it systematically concerned a means of communication (e.g. a kind of circumlocution to explain to someone which tool you are looking for). The rarity of pantomime in daily life suggests that it is not a routine, but rather an improvized and creative act,^34^^,^^43–46^ and this is not inconsequential. The gesture-engram hypothesis does not ignore that pantomime induces additional cognitive or motor-control demands compared to real tool use^15^^,^^38^ (e.g. the absence of external cues). However, the creative nature of pantomime questions the idea that there is a strict correspondence between the errors committed during pantomime and those committed during real tool use (e.g. posture errors could have distinct cognitive origins in pantomime versus real tool use).

The communicative hypothesis highlights that the function of pantomime is to inform observers about the manner of using a tool. In this vein, Goodglass and Kaplan^34^ proposed a scoring system describing communicative responses during pantomime [e.g. gestural enhancement, vocal overflow, body-part-as-object, hereafter called body-part-as-tool (BPT), based on our definitions of the terms tool and object, see Table  1]. Except the BPT responses that have since become popular, this proposal has not been adopted even by the proponents of the communicative hypothesis themselves. Yet, this description is very close to the clinical reality and reflects relatively well the individuals’ engagement in this communicative act, which they may perceive as a mime game. The communicative function of pantomime becomes even more obvious when we move from neuroscience to anthropology. Many attempts have been made to elucidate the adaptive value of pantomime in the human lineage and its potential co-evolution with tool-use skills.^47–50^ Pantomime has been suggested to be an early form of teaching (i.e. proto-language^48^) that could have been useful to transmit technical content and thus favour the emergence of cumulative technological culture in our lineage.^32^^,^^51^^,^^52^ Teaching can be defined as behaviour that facilitates learning in others.^53^ Teaching—or more precisely direct active teaching^53^—can take the form of verbal explanation or pointing movements. The teacher can also repeat or slow down the sequence of actions, or provide a demonstration punctuated by exaggerated movements.^53^ This latter aspect is reminiscent of the exaggerated amplitude of movements commonly reported during pantomime.^9^^,^^54^^,^^55^ Some have explored whether this exaggeration could result from the lack of external cues and feedback,^56–61^ without providing conclusive evidence for this. Others have already stressed that this exaggeration could be viewed as an attempt to facilitate the recognition of the tool-use action by the observers.^9^ In broad terms, this confirms that pantomime is a communicative act, which is improvized and creative. The emphasis put here on the communicative and creative aspects may appear exaggerated, but we argue that it is not: It might be the only way of envisioning an appropriate interpretation of pantomime. Pantomime must not be merely conceived as a real tool-use task minus some components (e.g. the possibility of holding the tool in hand or of performing a real mechanical action). The absence of these components imposes specific demands on individuals, who must decide, more or less consciously, which demonstration is expected (e.g. cutting or peeling with a knife) and which movement parameters increase the recognition of their demonstration by observers (e.g. arm amplitude). This is not to say that pantomime does not share any cognitive component with real tool use. Instead, this emphasis allows us to keep in mind the specificity of pantomime compared to real tool use, which might be essential to apprehend the neurocognitive bases of pantomime. Before discussing in more details the dissimilarities between pantomime and real tool use, let us begin by their commonalities.

## Pantomime and real tool use: the left area PF

**Figure 1:**
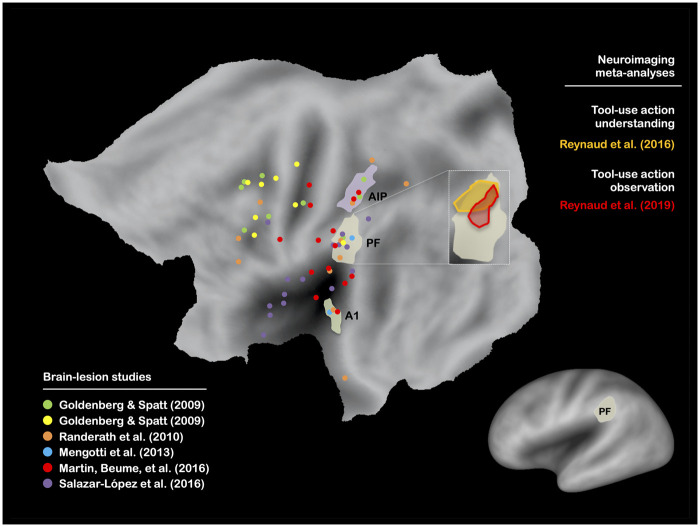
**Brain-lesion studies of real tool use. Maximum lesion overlap locations are represented on the PALS-B12 left hemisphere (flat map, main figure; very inflated map, mini figure) atlas surface configuration.**^249^ Brain areas are identified using anatomical labels from the parcellation of Glasser et al.^81^ Only the brain areas that have been reported in at least 50% of the conditions included here are represented (see Supplementary Methods). The main findings obtained by two neuroimaging meta-analyses on tool-use action understanding^111^ and tool-use action observation^114^ are also shown.

**Figure 2:**
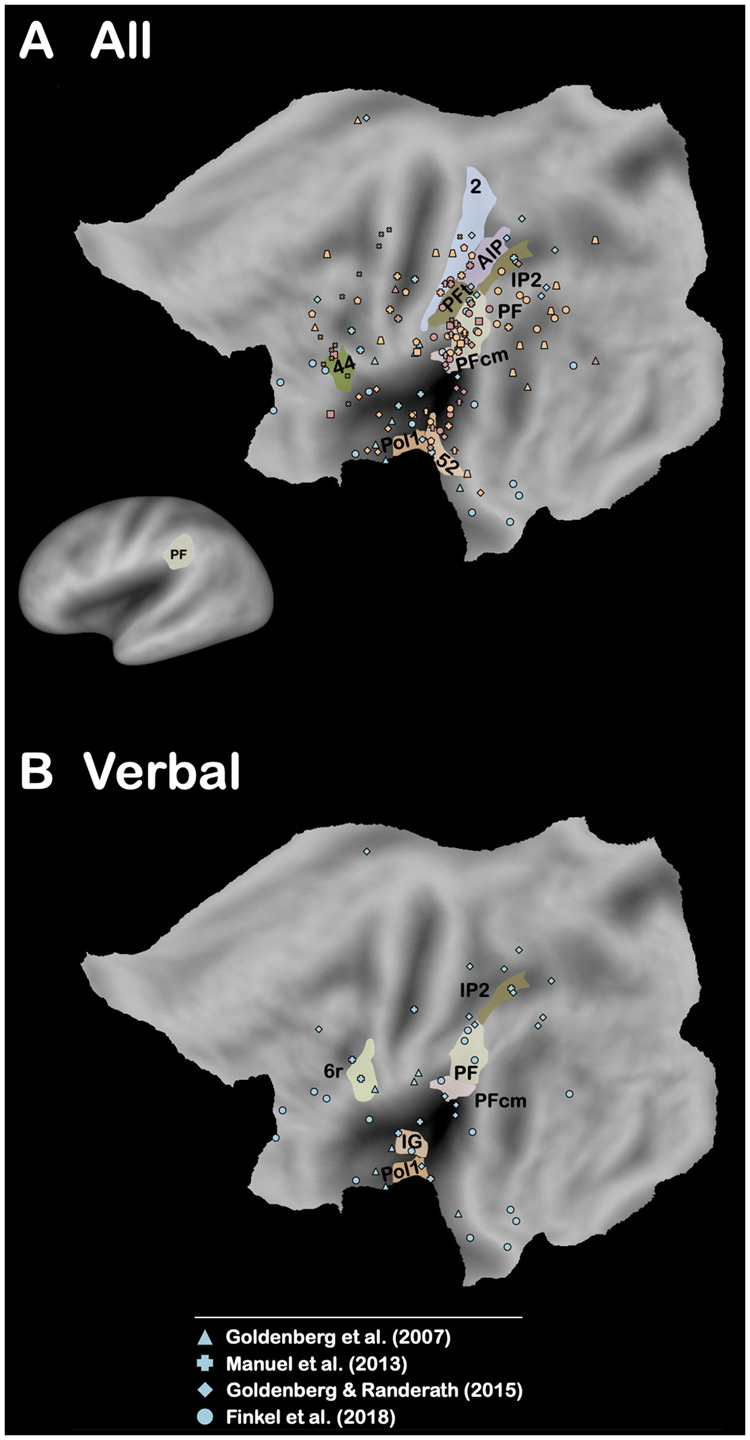
**Brain-lesion studies of pantomime of tool use (All and Verbal).** Maximum lesion overlap locations are represented on the PALS-B12 left hemisphere (flat maps, main figures; very inflated map, mini figure) atlas surface configuration.^249^ Brain areas are identified using anatomical labels from the parcellation of Glasser et al.^81^ (**A**)Only the brain areas that have been associated with the three modalities (verbal, visual and imitation) are represented. (**B**) Only the brain areas that have been reported in at least 50% of the conditions included for the verbal modality are represented (see Supplementary Methods).

**Figure 3:**
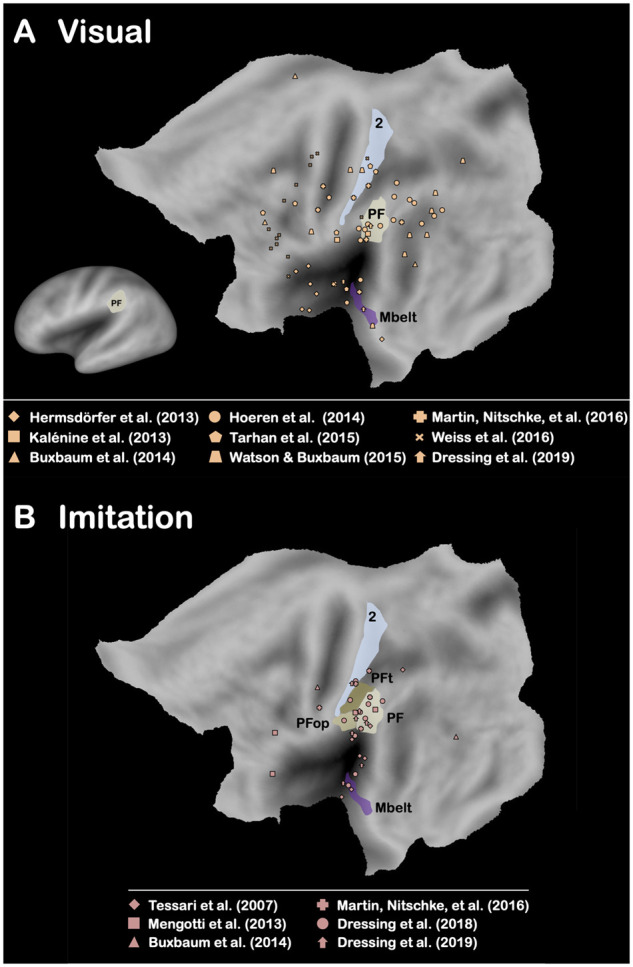
**Brain-lesion studies of pantomime of tool use (Visual and Imitation).** Maximum lesion overlap locations are represented on the PALS-B12 left hemisphere (flat maps, main figures; very inflated map, mini figure) atlas surface configuration.^249^ Brain areas are identified using anatomical labels from the parcellation of Glasser et al.^81^ (**A**) Only the brain areas that have been reported in at least 50% of the conditions included for the visual modality are represented. (**B**) Only the brain areas that have been reported in at least 50% of the conditions included for the imitation modality are represented (see Supplementary Methods).

**Figure 4:**
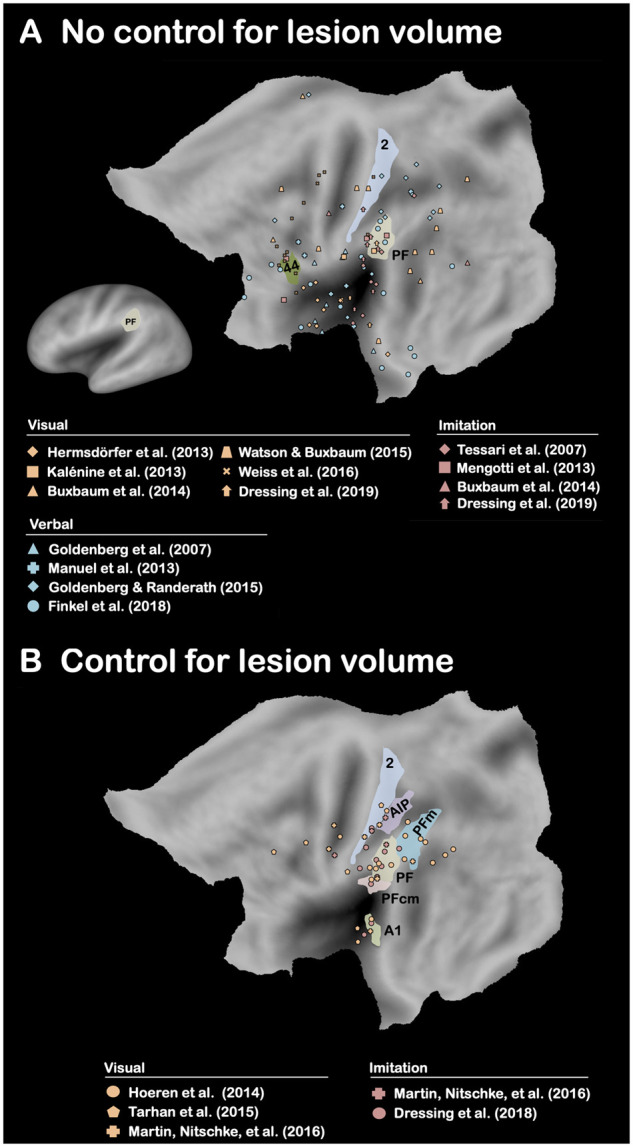
**Brain-lesion studies of pantomime of tool use (No control and Control for lesion volume).** Maximum lesion overlap locations are represented on the PALS-B12 left hemisphere (flat maps, main figures; very inflated map, mini figure) atlas surface configuration.^249^ Brain areas are identified using anatomical labels from the parcellation of Glasser et al.^81^ (**A**) Only the brain areas that have been reported in at least 50% of the conditions in which there was no control for lesion volume are represented. (**B**) Only the brain areas that have been reported in at least 50% of the conditions in which there was a control for lesion volume are represented (see Supplementary Methods).

A significant body of evidence has indicated a strong association in left brain-damaged (LBD) patients between performance in pantomime and real tool use, when real tool use is assessed by asking patients to use either familiar (i.e. familiar tool use,^15^^,^^58^^,^^62–71^ for an association between single tool use and familiar tool use, see De Renzi and Lucchelli^6^, Neiman et al.^58^ and Buchmann and Randerath^72^) or novel tools.^66^^,^^67^^,^^72–75^ This association has also been corroborated by kinematic analyses.^54^^,^^55^^,^^61^ The question is whether this behavioural association reflects shared neural substrates between pantomime and real tool use. To address this question, we present the results from the five brain-lesion studies^76–80^ (six conditions) that have explored real tool use in LBD patients (familiar tool use, *n *=* *5; novel tool use, *n *=* *1; Supplementary Table 1). As shown in Fig.  1, the only brain area that is systematically associated with impaired performance in real tool use is the left area PF^81^ (Area PF complex) within the left IPL. We also report the results from the 16 brain-lesion studies^9^^,^^16–19^^,^^25^^,^^26^^,^^30^^,^^31^^,^^78^^,^^82–87^ (19 conditions) that have investigated pantomime in LBD patients (verbal modality, *n *=* *4; visual modality, *n *=* *9; imitation modality, *n *=* *6; Supplementary Table 2). The results are less straightforward than for real tool use because no brain area is systematically associated with impaired performance (Figs  2–4). This indicates that pantomime is more multidetermined cognitively than real tool use. We will come back to this crucial point below. Importantly, this analysis also reveals that only the left area PF is involved whatever the modality (Fig.  2A) as well as in at least 50% of the studies when each modality is taken separately (Figs  2B and 3A and B). The same conclusion is drawn when the studies are divided into those controlling for lesion volume or not (Fig.  4A and B). This finding is consistent with the long tradition in neuropsychology, which has repeatedly stressed the involvement of the left IPL, containing the area PF, in pantomime (but see Refs.^8^^,^^23^^,^^82^^,^^88–90^).

**Figure 5:**
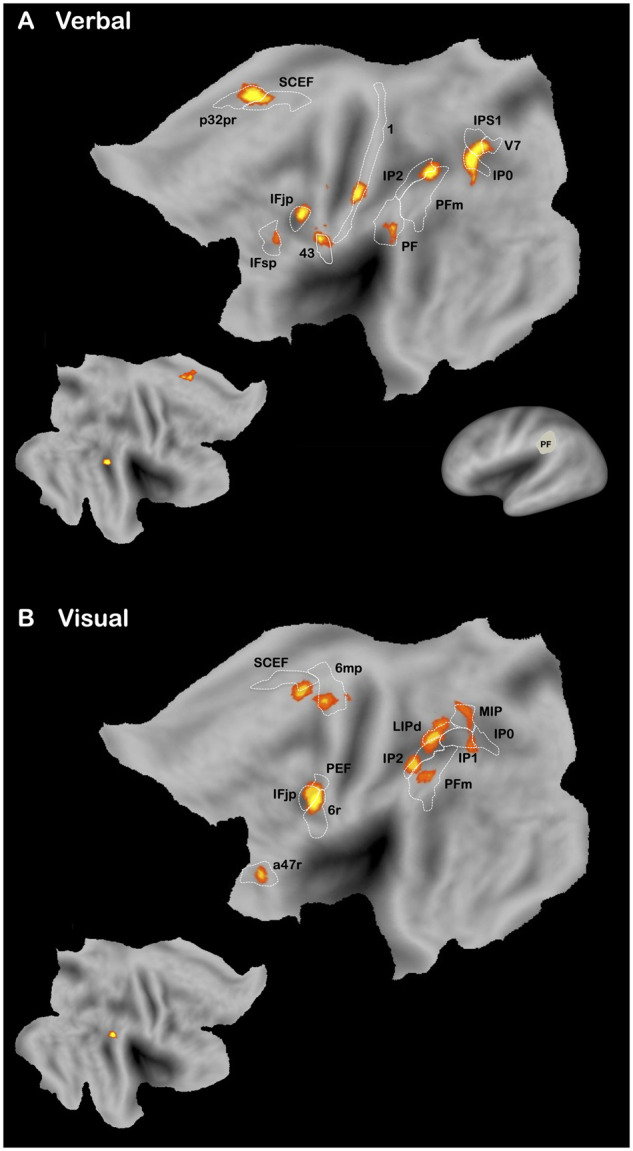
**Neuroimaging studies of pantomime of tool use (Verbal and Visual).** ALE maps are represented on the PALS-B12 left (main figures) and right (mini figures) hemisphere atlas surface configurations.^249^ Brain areas are identified using anatomical labels from the parcellation of Glasser et al.^81^ (see Supplementary Methods).

We conducted an additional meta-analysis of neuroimaging studies on pantomime^23^^,^^37^^,^^91–107^ (verbal modality, *n *=* *10; visual modality, *n *=* *11; Supplementary Table 3) to explore whether this finding can be extended to healthy participants. As shown in Fig.  5, two brain areas were activated in both modalities: The left intraparietal sulcus (IPS; IP0, IP2, LIPd, MIP) and the left inferior frontal gyrus (IFG; IFJp). The left area PF is also involved in the verbal modality but not in the visual modality. We do not have any satisfactory interpretation for this discrepancy (e.g. difference in terms of ‘processing depth’ between the two modalities). Therefore, we will not elaborate further on this point, which nevertheless temper our conclusions. Regardless, these results partly confirm that the left area PF is activated when healthy participants produce pantomime.

The communicative hypothesis is not a good candidate to account for the central role of the left area PF in both pantomime and real tool use as well as the behavioural association between both tasks. This hypothesis holds that communicative/language skills are critical to pantomime but not to real tool use. Therefore, the neural bases of pantomime should mainly concern the cortical structures involved in communication/language skills [i.e. ventral structures such as temporal lobes (TL)], and no strong behavioural association should be found between pantomime and real tool use. Concerning the former point, it is noteworthy that most of LBD patients have middle cerebral artery lesions, which impact several brain areas involved in language skills (e.g. superior temporal sulcus or the left IFG) but also spare the polar portions of the temporal cortex that are known to play a key role in communicative skills. As a result, in LBD studies, the role played by communicative skills could be potentially underestimated. We will come back later to this point.

The gesture-engram hypothesis may appear more likely to explain these findings, in suggesting that the central role of the left area PF in both pantomime and real tool use and their behavioural association reflect the involvement of gesture engrams. Remind that gesture engrams refer to motor programs containing information about *hand–tool* relationships (i.e. motor actions) for the use of *familiar* tools. However, the evidence reported above does not provide support for this interpretation.

First, the behavioural association between pantomime and real tool use in LBD patients has been found not only for familiar tool use but also for novel tool use. Brain-lesion studies also indicate that the left area PF is associated with both familiar and novel tool use (evidence also indicates a strong behavioural link between familiar tool use and novel tool use^66^^,^^67^^,^^73^^,^^108–110^). This is also consistent with evidence showing that a similar cerebral network is recruited when healthy participants pantomime both familiar and novel tools.^102^ However, gesture engrams are thought to be involved only in the real use or pantomime of familiar tools so that no strong link should be reported between the real use or pantomime of both familiar and novel tools. Therefore, the gesture-engram hypothesis is inappropriate to account for these findings, which corroborate that pantomime is an act that is improvized based on the current situation (i.e. familiar or novel).

Second, the gesture-engram hypothesis suggests that patients with tool-use disorders meet difficulties in executing the appropriate motor actions to use familiar tools. However, another interpretation is that these patients execute the motor actions that are *appropriate* to realize the *inappropriate* mechanical action (i.e. tool–object relationship) they intend to perform. The problem is that the pantomime task is not suited to disentangle between these two interpretations because only the motor action can be observed. Thus, it is impossible to determine whether the difficulty concerns the representation of the motor action or of the mechanical action. Interestingly, real tool-use tasks can also assess the ability to select appropriate tools and objects (i.e. tool selection). Impaired tool selection cannot be interpreted in terms of motor-engram deficits, since the individual does not have to perform the motor action associated with the use during this selection. Instead, impaired tool selection necessarily reveals difficulties in understanding the appropriate mechanical action to realize. This is all the more true for novel tool-use tasks in which individuals are asked to select and even sometimes make novel tools to solve mechanical problems. Therefore, the behavioural association found between pantomime and real tool use (both familiar and novel tool use) seems to indicate difficulties in forming an accurate representation of the mechanical action to be performed, even when no tool is held, or no mechanical action is concretely performed, as in pantomime.

This interpretation is also consistent with two recent meta-analyses of neuroimaging studies on tool use. The first meta-analysis^111^ included studies in which healthy participants had to focus on the appropriateness of either the motor action (e.g. hand posture-tool; called GESTURE in this meta-analysis; no pantomime studies included) or the mechanical action (e.g. orientation of the tool in function of the object; called ACTION in this meta-analysis; no pantomime studies included). The findings indicate that the IPS was preferentially activated for motor actions, and the left area PF for mechanical actions (Fig.  1). This finding clearly challenges the gesture-engram hypothesis, which suggests that the left IPL and not the IPS is central to gesture engrams.^20^^,^^93^^,^^112^^,^^113^ The second meta-analysis^114^ included studies on non-tool-use action observation (i.e. observing someone grasping an object) and tool-use action observation (i.e. observing someone using a tool with an object). The results confirmed the now classical fronto-parieto-temporal action observation network.^115–117^ More relevant to our concerns, the contrast tool-use action observation minus non-tool-use action observation revealed a specific activation of the left area PF (Fig.  1). Again, this highlights that this brain area is involved in the processing of mechanical actions between tools and objects, since the presence of mechanical actions was the only difference between the two types of action observation.

Taken together, these findings suggest that the left area PF is central for the processing of mechanical actions irrespective of whether the tool-use action is familiar or novel. This invalidates former interpretations in terms of gesture engrams, since these engrams are thought to contain information about motor actions for familiar tool use. The idea that an impaired processing of mechanical actions can disturb pantomime may sound odd, since the only aspect of the action that we can observe is the motor action. Nevertheless, this is consistent with the ideomotor principle,^118–122^ according to which motor actions are necessarily guided by the realization of an expected effect. In the case of pantomime, this perceptual effect is the mechanical action even if this mechanical action is performed with tools and objects that are nevertheless absent. We will discuss this aspect in more details below.

## Technical reasoning

Technical reasoning is a causal and analogical reasoning, which is directed towards the physical world.^33^^,^^123–127^ It is based on mechanical knowledge, which contains information about physical principles/mechanical actions. Technical reasoning is much more than spatial reasoning, which can consist, for example, in determining whether a car can pass between two trees or whether two puzzle pieces can be arranged together. It also involves the material dimension of physical objects (e.g. sharpness, hardness), which is the prerequisite for the understanding of physical principles/mechanical actions (e.g. lever, cutting). In this respect, people reason technically as soon as they must perform ‘mental making’, either by using tools but also by making them or building constructions.^128^

The function of technical reasoning is to generate technical solutions (i.e. potential mechanical actions) in selecting the appropriate tools and objects to solve physical problems, which can be familiar (e.g. to cut an apple) or novel (e.g. to get a ball that rolled under a couch). The outcome of technical reasoning is a mental simulation of the mechanical action to be performed (e.g. the motion of a knife on an apple). However, it does not deal with the selection and on-line control of the most appropriate motor actions to realize the mentally generated mechanical action. This is the function of the motor-control system, which is blind to the goal of the action (i.e. tool use or object transport). If someone has the idea of performing back-and-forth movements with a knife on an apple, this is the expected effect, which constrains the motor actions chosen by the motor-control system. Likewise, if someone intends to move an object from one location to another, the expected effect is the motion of the object, which constrains the motor actions chosen. In broad terms, the technical-reasoning hypothesis is akin to the ideomotor principle, and assumes that no specific tool-use motor programmes (i.e. gesture engrams) are required to specify how to manipulate familiar tools.^3^

The left area PF is the key neural substrate of technical reasoning, which is consistent with its involvement in any tool-use situations (e.g. familiar tool use, novel tool use), including pantomime, as well as in situations in which people must reason about physical events. In a way, technical reasoning can be viewed as a kind of ‘mechanical imagery’ (and not of spatial imagery, which is restricted to spatial reasoning; see above). By contrast, the motor-control system is supported by more superior structures within the dorso-dorsal stream (e.g. IPS) and is recruited for the processing of motor actions (i.e. motor imagery), as evidenced in the aforementioned meta-analysis of neuroimaging.^111^ Thus, damage to the dorso-dorsal stream can lead to impaired processing of motor actions, as described in specific forms of apraxia (e.g. motor apraxia). However, the technical-reasoning hypothesis posits that the nature of the difficulties met by LBD patients in pantomime and real tool use after damage to the left area PF is more technical because it concerns the understanding of mechanical actions. In this respect, the term *atechnia*^129–131^ may better reflect these difficulties (see below).

The technical-reasoning hypothesis shares some resemblance with other accounts that have suggested the existence of a potential non-lexical route between object structure and their potential use^66^^,^^67^^,^^132^ (e.g. affordance, inference of function from structure). However, it differs crucially from them, in assuming that technical reasoning is involved in any situation that needs mental making, including the use of physical tools. Thus, technical reasoning is not limited to novel tool use or a kind of compensatory strategy when gesture engrams are impaired.^67^^,^^72^^,^^79^^,^^112^^,^^133–136^ It is also not a synonym with ‘structural affordance’, which concerns the ability to extract a potential motor action (e.g. power or precision grip) from the mere observation of the structure of an object.^24^^,^^137–139^ For the technical-reasoning hypothesis, this is the specific role of the motor-control system (i.e. egocentric, hand–tool relationships or hand–object relationships in the case of a non-tool-use action such as object transport). Indeed, structural object properties can also be processed to generate mechanical actions between external objects (i.e. allocentric, tool–object relationships). In this case, it involves technical reasoning, even when the external object is the body of the user itself (e.g. brushing teeth with a toothbrush).

## Kinematics: motion of the tool not of the body

The kinematic component of pantomime refers to the global shape of the movement trajectory. This component is commonly considered as critical^140^ because this is what makes a demonstration recognizable or not by observers. Thus, when a quantitative approach is used (e.g. 2, 1 or 0 point for each pantomime), the quality of kinematics is roughly associated with the number of points given (i.e. from recognizable to unrecognizable^34^^,^^67^^,^^141^). The gesture-engram hypothesis posits that the kinematics component is contained within gesture engrams, so impaired gesture engrams are responsible for the production of motor actions characterized by incorrect kinematics. Although this proposal is viable at a theoretical level, the evidence described above against it leads us to envisage another interpretation.

**Figure 6:**
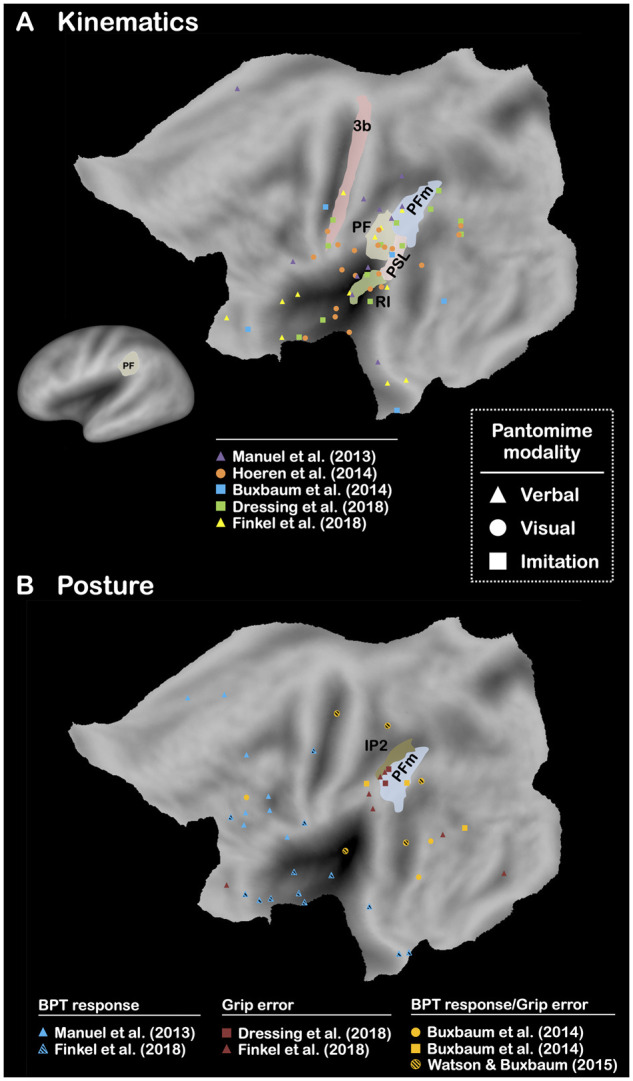
**Brain-lesion studies of pantomime of tool use (kinematics and posture components).** Maximum lesion overlap locations are represented on the PALS-B12 left hemisphere (flat maps, main figures; very inflated map, mini figure) atlas surface configuration.^249^ Brain areas are identified using anatomical labels from the parcellation of Glasser et al.^81^ (**A**) Only the brain areas that have been reported in 50% of the conditions included are represented. (**B**) Here, the 50% refers to either BPT response and BPT response/grip error (*n *=* *5 studies), or grip error and BPT response/grip error (*n *=* *5 studies; see Supplementary Methods).

The technical-reasoning hypothesis predicts that the mental simulation of the mechanical action guides motor actions during pantomime (i.e. ideomotor principle). In other words, it is the kinematics of the mechanical action that constrains the kinematics of motor actions needed to realize the mechanical action given. Therefore, given that the generation of mechanical actions could be mainly supported by the left area PF, this brain area should be preferentially involved in the kinematic component of pantomime. We tested this prediction by exploring the results of five brain-lesion studies,^16^^,^^17^^,^^25^^,^^31^^,^^85^ which have investigated the lesion sites associated with kinematic errors (verbal, *n *=* *2; visual, *n *=* *1; imitation, *n *=* *2; Fig.  6A; Supplementary Table 4). As shown, only the left area retroinsular cortex and the left area PF were reported in four of the five studies. In broad terms, as suggested by the technical-reasoning hypothesis and the ideomotor principle, the left area PF could be responsible for the processing of mechanical actions (i.e. motion of the tool) rather than of motor actions (i.e. motion of the body).

## Posture: an experimental artefact?

The posture component (i.e. grip) is considered as the second key component of pantomime. The neural bases associated with posture errors have been subject to debate, even by the same research groups, who have found discrepancies over time (e.g. left IPL^112^^,^^142^; Left IPL and IPS^13^; left posterior temporal gyrus^17^; left IPL^19^). In Fig.  6B, we present the results of four brain-lesion studies^17^^,^^19^^,^^31^^,^^85^ in LBD patients (five conditions) that have investigated the lesion sites associated with posture errors, and more particularly with either grip errors or grip errors/BPT responses (verbal, *n *=* *1; visual, *n *=* *2; imitation, *n *=* *2; Supplementary Table 4). The pattern of results corroborates the aforementioned discrepancies, with no clear lesion sites except perhaps the IPS, which is found in both studies on grip errors only. Although this pattern of results must be considered with caution given the low number of studies (*n *=* *4), it contrasts with the more straightforward results obtained for the kinematic component. This also questions the gesture-engram hypothesis or at least some of its version, which predicts an association between posture errors and the left IPL or temporal structures. At best, it can be hypothesized that the posture component is supported by the IPS, which would be consistent with its role in motor control and grip planning. However, the discrepancy of these results also leads us to consider another interpretation.

An intriguing aspect of the posture component is that posture errors found during pantomime are rarely reported in LBD patients in real tool use. Indeed, only few cases of patients have been reported with severe posture errors in real tool use (e.g. grasping a tool upside-down),^71^^,^^143^^,^^144^ which contrasts with the frequent occurrence of posture errors found in the pantomime tasks. Posture errors (e.g. grasping a tool upside-down) can increase when LBD patients are asked to actually grasp a tool (single tool use), while the tool is presented with the handle pointing away from the patient’s body^145^ (note that such errors can also be observed in healthy young participants under dual-task conditions^146^). However, these errors disappear dramatically in real tool-use tasks.^71^ In broad terms, the posture errors reported in pantomime cannot be viewed as an indicator of posture errors in real tool use, which suggests that pantomime is not a strict proxy for studying real tool-use disorders. Additional evidence supports this conclusion. Indeed, it has been shown that the most significant characteristics of a natural prehension movement are diminished or absent during pantomime of grasping movements.^147–149^ At best, hand aperture matches or indicates the diameter of the object grasped but does not represent the real motor action.

These findings stress that individuals meet difficulties in performing the posture component during pantomime. One potential interpretation is that it is cognitively demanding. Another non-exclusive interpretation that is in line with the communicative hypothesis is that the posture component is not crucial for pantomime, because its recognition is mainly based on the kinematic component. Interestingly, both individuals and examiners seem to share the same belief: To be recognizable—or to obtain a higher score—it is better to favour the kinematic component. This is consistent with the goal-directed imitation model,^150^ which has been initially developed for action imitation. According to this model, the individual does not imitate a movement as a whole but rather decomposes it into its separate aspects. Then, these aspects are hierarchically ordered, and the highest aspect becomes the individual’s main goal. The corollary is that the lowest aspects are not necessarily well produced, particularly if there is no sufficient cognitive resource. This model has already been discussed in the literature on imitation of meaningless postures.^151^^,^^152^ Here we propose to extend it to the case of pantomime. Thus, if we consider that (i) pantomime is a communicative act, (ii) the kinematic component is the highest aspect for recognition and (iii) pantomime is a creative, cognitively demanding act, then this can explain why the posture component is not favoured during pantomime. As a result, it is easier to understand why it is difficult to identify its neural bases.

## Body part as tools

As mentioned above, BPT responses were initially described by Goodglass and Kaplan^34^ as a kind of communicative responses. Since, there has been an intense debate as to whether BPT responses are pathognomonic of apraxia.^153–156^ The main reason is that BPT responses can be found in many pathologies that are not characterized by real tool-use disorders [e.g. autism^157^^,^^158^; Schizophrenia^159–161^; but also after right brain damage^42^^,^^153^^,^^155^ (RBD)]. More intriguingly, healthy participants can also produce BPT responses^31^^,^^34^^,^^153^^,^^162^ and even sometimes in high proportion^163^ (e.g. 30%). Heilman and Rothi^10^ stressed that, when a BPT response occurs, it is fundamental to reinstruct the individuals that they have to pretend to hold the tool in hand, and not to shape the hand as if it was the actual tool.^13^^,^^154^^,^^164^ It is only when the individual does not modify the response that a BPT response becomes pathologic. It is true that the instructions given are crucial because they can modulate the occurrence of BPT responses^163^ and that reinstructing the individual can lead to a decrease of BPT responses in healthy partcipants.^154^^,^^164^ However, reinstructing LBD patients with tool-use disorders does not guarantee that they understand the nuance given the high co-occurrence of aphasic deficits in these patients. Regardless, the fact that healthy participants—when not reinstructed—perform BPT responses confirms the communicative nature of pantomime, the individuals spontaneously attempting to produce a demonstration that can be easier to be recognized by an observer. In this respect, BPT responses should not be viewed as an artefact or errors to be controlled to make pantomime a better proxy for studying real tool-use disorders. Instead, they should be considered as an intrinsic aspect of pantomime, which reflects the interpretation made by the individual of the goal of the task: To communicate.

In this line, a key prediction is that it should be difficult to identify a specific neural basis for BPT responses from brain-lesions studies, because they do not characterize a particular deficit. To test this prediction, we report the results of the four brain-lesion studies^16^^,^^17^^,^^19^^,^^31^ (five conditions) that have explored the neural bases of either BPT responses or grip errors/BPT responses (verbal, *n *=* *2; visual, *n *=* *2; imitation, *n *=* *1; Fig.  6B; Supplementary Table 4). As shown, no specific lesion site is associated with BPT responses, corroborating that BPT responses are not pathognomonic of impaired pantomime.^153^ A potential interpretation of these findings is that BPT responses heavily depends on the nature of the tools presented, some of them clearly favouring the occurrence of BPT responses^31^^,^^153^^,^^165^ (e.g. scissors). Thus, the presence of such tools could be insufficient or not controlled, which can prevent from creating a good measure of these responses and, therefore, to obtain a significant link between them and lesion sites. One of the studies discussed here controlled for this aspect and found an increased frequency of BPT responses after damage to left ventral structures^31^ (i.e. inferior frontal cortex and anterior temporal cortex). For the authors, this confirms the link between BPT responses and communicative skills. Although we agree that BPT responses can be a manifestation of the communicative nature of pantomime, the conclusion drawn by the authors is unclear, because it suggests that impaired communicative skills increase the frequency of communicative responses. Predicting the inverse relationship sounds more viable at a theoretical level, namely, less BPT responses after damage to the cerebral structures underlying communicative skills. This illustrates the difficulty in interpreting the neurocognitive origins of BPT responses.

## Motor control and body schema

**Figure 7:**
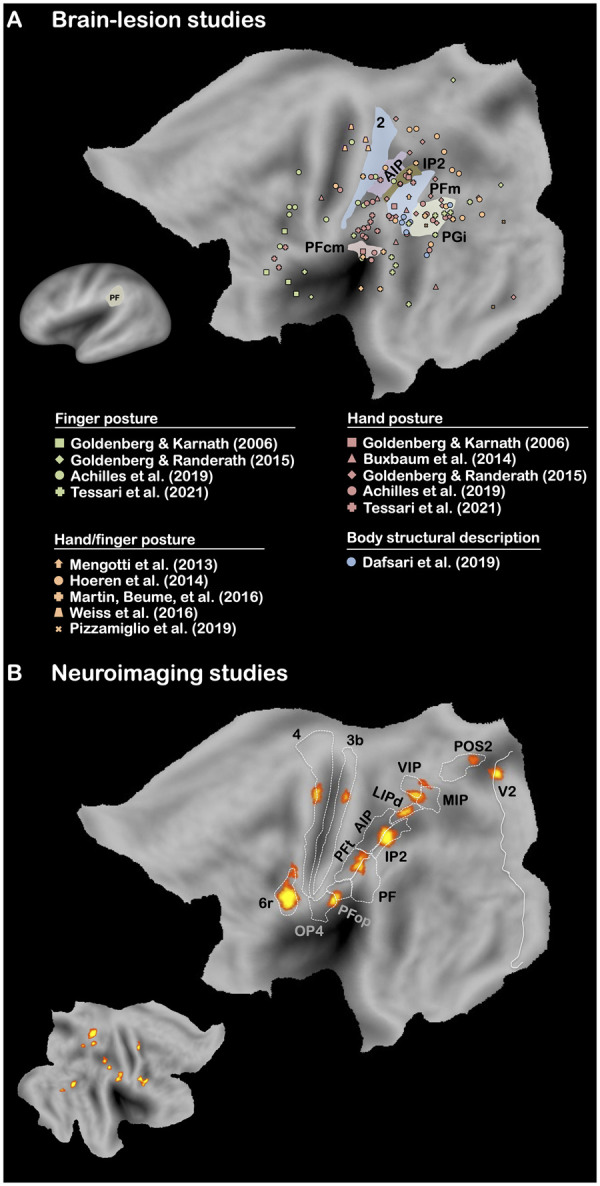
**Brain-lesion and neuroimaging studies of imitation of meaningless postures.** Maximum lesion overlap locations and ALE maps are represented on the PALS-B12 left (main figures; very inflated map, mini figure) and right (mini-figure) hemisphere atlas surface configurations.^249^ Brain areas are identified using anatomical labels from the parcellation of Glasser et al.^81^ For brain-lesions studies, only the brain areas that have been associated with the three types of posture (hand/finger, hand, finger) are represented (see Supplementary Methods).

Planning and on-line control of motor actions are supported by the motor-control system, which involves dorso-dorsal structures and notably the IPS.^166^ Planning and controlling motor actions need to dynamically keep track of the spatial relations between different body parts,^167–169^ which is referred to as body schema.^170^^,^^171^ Motor actions are obviously not directed only towards tool-use actions and can also concern non-tool-use actions (e.g. grasping or meaningless gestures). Therefore, deficits of the motor-control system/body schema should disturb both tool-use and non-tool-use tasks (e.g. imitation of meaningless postures). The general role of the motor-control system/body schema for action has been repeatedly stressed in the literature on apraxia^25^^,^^106^^,^^112^^,^^125^^,^^172–175^ on the basis of evidence indicating a behavioural association between pantomime and imitation of meaningless postures in LBD patients.^12^^,^^13^^,^^65^^,^^72^^,^^176^ This association is nevertheless not systematic.^6^^,^^177^^,^^178^ As shown in Figs  2–4, lesions to some IPS areas seem to be associated with pantomime performance. Results of neuroimaging studies are more straightforward, in stressing a clear activation of IPS during pantomime (Fig.  5). In order to explore this aspect in more details, we report the results of the 10 brain-lesions studies^17^^,^^25^^,^^26^^,^^30^^,^^78^^,^^84^^,^^179–182^ (14 conditions) that have investigated the lesion sites associated with imitation of meaningless postures (Hand/finger postures, *n *=* *5; Hand postures, *n *=* *5; Finger postures, *n *=* *4; Fig.  7A; Supplementary Table 5). As shown, we found only two studies in which the left area PF was found to be associated with impaired imitation,^180^^,^^182^ confirming its specific role for tool use. This also invalidates the idea that real tool use and imitation of meaningless postures strictly requires the same neurocognitive skills (i.e. categorical apprehension of spatial relationships).^3^^,^^183^^,^^184^ More particularly, the results highlight the involvement of the left angular gyrus (AG; PFm, PGi), which we will discuss in more details in the next section, and of the left IPS (AIP, IP2). In broad terms, these results confirm that the control-motor system is involved in both tool-use and non-tool-use actions (i.e. object transport, imitation of postures).

## Body structural description

Body schema is dedicated to the processing of motor actions that an individual can perform to interact with the physical world. However, the body can also be represented as an external object. These body representations can correspond to either lexical-semantic representations of the body (e.g. names, functions or relations with familiar tools; i.e. body semantics) or to a topological map of locations that defines body part boundaries and proximity relationships^170^^,^^171^ (i.e. body structural description). Here, we will focus on this latter form of body representations. Patients with impaired body structural description meet difficulties in pointing accurately to body parts either on their own body or on the examiner’s body/manikin. This deficit is commonly called autotopoagnosia. In imitation of meaningless postures, the individual is sometimes asked to perform postures towards the body. Thus, some of the errors committed can reflect difficulties in pointing accurately to body parts. In other words, imitation of meaningless postures could need body structural description. This possibility has repeatedly been suggested in the literature.^179^^,^^184–189^ Evidence also stresses that the LBD patients who are impaired to imitate meaningless postures also encounter difficulties in reproducing these postures on a manikin.^185^ In other words, imitation of meaningless postures could be based on both body schema and body structural description.

Interestingly, several studies have reported that impaired imitation of meaningless postures could be observed after damage to the left AG.^87^^,^^174^^,^^177^ This is corroborated by the results reported above, which also point out that the left AG is associated with difficulties in imitation of meaningless postures in LBD patients (Fig.  7A). Thus, the left AG could be strongly involved in body structural description. This prediction is confirmed by a recent study,^190^ which demonstrated the co-occurrence of autotopoagnosia and apraxia as well as the involvement of the left AG in the task assessing body structural description (Fig.  7A).

To investigate further this point, we conducted a meta-analysis of neuroimaging studies on the 13 studies^152^^,^^191–202^ (19 conditions) that have explored imitation of meaningless postures (Hand/finger postures, *n *=* *1; Hand postures, *n *=* *5; Finger postures, *n *=* *13; Fig.  7B; Supplementary Table 6) for a similar analysis on both meaningless and meaningful gestures.^203^ The results indicate a relatively symmetric and bilateral network, which contrasts sharply with the left lateralization observed for tool-use actions^111^^,^^114^ including pantomime. Importantly, there was a clear involvement of the IPS (AIP, IP2, LIPd, VIP, MIP), which corroborates the role of the motor-control system/body schema in imitation of meaningless postures. However, no preferential activation of the left AG was reported, contrary to what we found for brain-lesion studies. This finding stresses the frequent discrepancy that can be reported between brain-lesion and neuroimaging studies^7^^,^^203^^,^^204^ (see also above for pantomime). Nevertheless, the discrepancy noted here could make sense and even help us better understand the neurocognitive bases of imitation of meaningless postures. Indeed, several authors have already stressed that neuroimaging studies can provide divergent results because of the experimental constraints imposed by the scanner.^16^^,^^82^^,^^99^ More specifically, the postures produced in the scanner usually correspond to hand or finger postures that are not directed towards the body or, at best, towards the supine trunk of the body,^191^^,^^195^ whereas in clinical testing postures are directed towards other body parts^82^ (e.g. mouth, face). This constraint could reduce the requirement of body structural description and explain the absence of activation of the left AG in neuroimaging studies.

Studies interested in pantomime generally do not distinguish between tool-use actions directed towards (e.g. toothbrush, comb) and away from the body (e.g. hammer, knife). However, tool-use actions directed towards the body could require the additional involvement of body structural description.^205–207^ This is consistent with a recent study, which showed that pantomiming a tool-use action directed towards versus away from the body involves distinct cognitive processes.^208^ Future research is needed to test this prediction, which could explain why the left AG has been sometimes found to be associated with tool-use disorders.^13^^,^^30^^,^^77^^,^^174^

## Semantic knowledge

Performance on pantomime tasks is difficult to measure,^209^ notably for a clinician who cannot systematically videotape it and ask help from a colleague for the scoring. This difficulty lies in the fact that an incredible number of productions can be performed with the same tool (e.g. a knife can be used to cut or peel an apple, or to spread butter), which do not necessarily correspond to the so-called prototypical production expected by the examiner.^131^^,^^210^^,^^211^ This is all the more true when only the name of the tool is given, without allowing the individual to see the real tool or have more information about the tool-use action expected. Again, this questions the idea that pantomime is a good proxy for studying real tool-use disorders. Interestingly, this variability in the production can also be viewed as very instructive, reminding us that pantomime puts additional demands on individuals, who must decide what is expected by the examiner, while it does not necessarily correspond to the way they usually use the tool in their daily life (e.g. pretending to read newspapers while the individual is not used to read them but to light fire with them). This is also true for single tool use, because even if, in this case, the tool can be grasped during the demonstration, the absence of the corresponding object prevents individuals from knowing which specific mechanical action is expected. In sum, to solve this problem, individuals must use their knowledge about the social usages associated with tools and objects, which refer to the function and the context of use of the tool that are globally shared by the members of the social group to which they—including the examiner—belong.

Knowledge about social usages associated with tools and objects refers to what is commonly called functional or contextual knowledge.^133^^,^^212^ We will use the more generic term semantic knowledge. This knowledge was once considered as crucial for the real use of familiar tools.^66^^,^^213^^,^^214^ However, a large body of evidence has demonstrated that this knowledge is neither necessary nor sufficient for real tool use.^215–219^ For instance, patients with semantic dementia, who are characterized by selective semantic deficits, perform in the same range as healthy matched controls on novel tool use,^133^^,^^220^^,^^221^ which corroborates that their technical-reasoning skills are preserved. The fact that, in semantic dementia, the atrophy mainly affects the polar portions of the TLs—and not the left IPL and, therefore, not the left area PF—is also consistent with this pattern. Patients with semantic dementia also perform relatively well in familiar tool-use tasks, when no choice is allowed (i.e. only the tool and its corresponding object).^220^ Nevertheless, because of semantic deficits, these patients can fail to recognize the tools and objects presented, putting them in a situation very close to a novel tool-use task. In fact, for them, familiar tool use may even be more difficult than novel tool use. Indeed, the instructions commonly given in familiar tool use is to ask the individual to show how to use a tool and an object together. Thus, no goal is provided. Instead, it must be inferred from the structure of the tool and the object provided. By contrast, in novel tool-use tasks, the goal is explicitly given by the examiner or can be understood more easily from practice trials, since it does not change during the task^133^^,^^221^ (e.g. to lift a cylinder; to extract a target out from a box). Thus, unexpected responses can be reported in patients with semantic dementia in no-choice conditions of familiar tool-use tasks. This is particularly likely when the mechanical action between a tool and its target is opaque. For instance, patients with semantic dementia can insert a key into a padlock without turning the key.^220^ It is not obvious to identify that a padlock possesses a hidden mechanical system by looking at it. In this case, semantic knowledge can be relevant in providing the information that it is something commonly used to lock. Such errors can also be found in choice conditions of familiar tool-use tasks, the patient being unable to identify what is the mechanical action expected.^220^ In a way, even if semantic knowledge is not a generator of mechanical actions—this is the function of technical reasoning—, it can nevertheless be helpful to decide which mechanical action is expected in a social context such as the clinical setting. In line with this, it is noteworthy that, in these patients and contrary to LBD patients with tool-use disorders, the difficulties observed in clinical tasks are not found in their daily lives, in which they can use their own tools and objects in order to achieve the goals they set themselves.^222^

**Figure 8:**
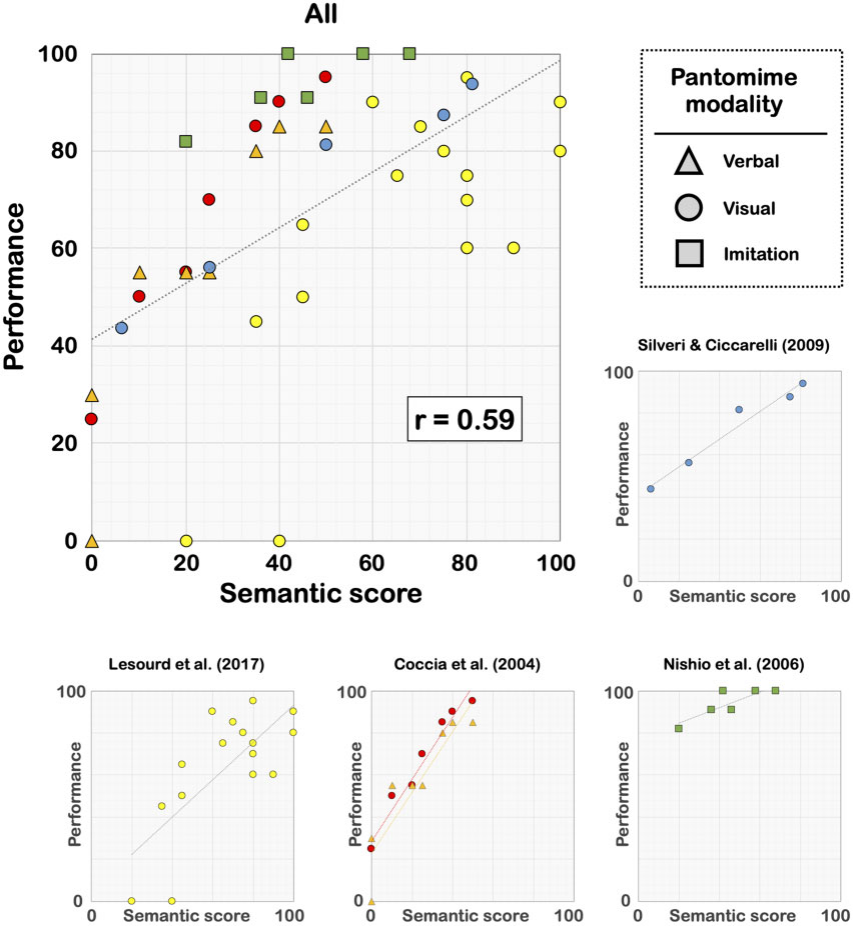
**Link between semantic knowledge and pantomime in patients with selective semantic deficits.** Performance was systematically converted into percentages. In the study of Coccia et al.,^225^ the eight points reported for each modality refer to two patients who were assessed over a 4-year longitudinal period. The coefficients *r* are Pearson correlation coefficients (see Supplementary Methods).

**Figure 9:**
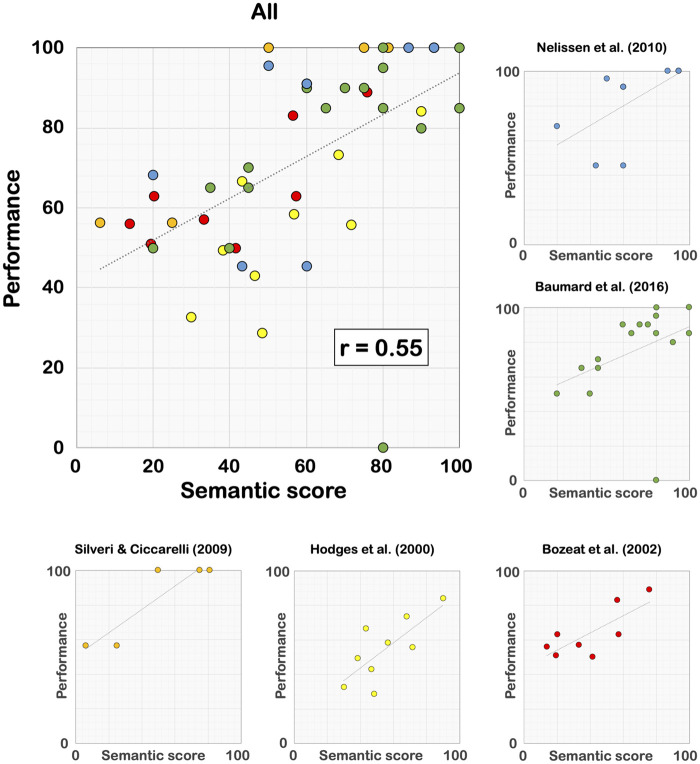
**Link between semantic knowledge and single tool use in patients with selective semantic deficits.** Performance was systematically converted into percentages. The coefficients *r* are Pearson correlation coefficients (see Supplementary Methods).

The difficulties in recognizing familiar tools can have an even greater deleterious effect when tools are presented in isolation, such as in pantomime or single tool use. As explained above, for a patient with selective semantic deficits, a familiar tool-use task can become a novel tool-use task, which can be more difficult to solve because of the absence of explicit goal. However, in pantomime or single tool use, the individual cannot infer at all the potential mechanical action expected given that no additional object is provided. Interestingly, in this case, the patient can use a specific strategy consisting in attempting to show how to use the tool presented in isolation with the objects available in the clinical context.^218^^,^^223^ Therefore, a key prediction is that patients with selective semantic deficits should be impaired in pantomime and single tool use, and that the performance in these tasks should be associated with the severity of semantic deficits. We explored these predictions by collecting the data from eight behavioural studies^133^^,^^211^^,^^219^^,^^220^^,^^224–227^ (Supplementary Table 7), in which patients with selective semantic deficits (i.e. semantic dementia or herpetic encephalitis) have been assessed on both semantic tasks and pantomime (*n *=* *4) or single tool use (*n *=* *5). In all these studies, patients performed worse than healthy matched controls, validating our first prediction. Our second prediction was also correct since a robust link was observed between the performance on pantomime/single tool use and the severity of semantic deficits (Figs  8 and 9). Interestingly, this link has also been reported in LBD patients.^66^^,^^73^ Taken together, these findings emphasize that semantic knowledge can play a crucial role in pantomime, in helping the individual identify the mechanical action that is ‘socially’ expected (for somewhat similar interpretations, see Randerath et al.,^15^ Hoeren et al.^25^ and Goldenberg and Randerath^30^).

## From language to communication

The communicative hypothesis has been sometimes confounded with the language hypothesis, which assumes that tool use and language might be based on common cognitive processes as suggested by the concept of asymbolia.^12^^,^^27^^,^^28^ It is known for a long time that the prevalence of apraxia is higher in patients with aphasia.^1^^,^^38^^,^^78^^,^^228^ In this vein, auditory comprehension tasks strongly correlate with pantomime.^30^^,^^68^^,^^109^ There is also evidence that tool use and language are co-lateralized and that this co-lateralization is independent of the manual dominance.^104^^,^^144^^,^^178^^,^^229^ However, the language network differs from the tool-use network, with no involvement of the left area PF.^230^ A recent study also showed that language and tool-use disorders involve different neural substrates.^79^ As a result, the most likely hypothesis is that the lesions responsible for tool-use disorders very frequently encroach on the brain areas specialized for language,^228^ creating an illusory correlation in many studies. As discussed above, semantic knowledge could be critically involved in pantomime. Thus, semantic knowledge could be the only linguistic component that is shared with tool use, and particularly, pantomime.

In the psychology and neuroscience literature, communication skills are generally considered as supported by theory-of-mind skills,^231^ which allow the individual to think about others’ mental states (i.e. perspective taking). Interestingly, in children, there is a co-evolution between pantomime and theory-of-mind skills.^232^ Pantomime is also impaired in some pathologies that are characterized by theory-of-mind deficits, such as autism spectrum disorders^157^^,^^158^^,^^233^^,^^234^ or schizophrenia.^160^^,^^161^^,^^235^ Indeed, as any communicative act, producing a pantomime requires taking the perspective of the observer. As explained above, if the pantomime concerns a familiar tool, semantic knowledge is needed to identify which is the associated social usage and, as a result, the demonstration that is the most likely to be recognized. If the tool is unfamiliar, it can also be needed to identify what is the most relevant to mime from the observer’s perspective. Regardless, in both cases, perspective taking is required. Interestingly, the theory-of-mind network includes the anterior portions of the TLs,^236^ thereby suggesting that semantic knowledge could be involved in theory of mind^236^ as suggested above. In this respect, this possibility could stimulate a renewed interest for some aspects of pantomime that have been so far mainly concerned as methodological problems. For instance, if the presence of exaggerated amplitude in pantomime is a characteristic shared by a great number of individuals (to make the demonstration easier to recognize), the question could be to investigate the cognitive specificity of the individuals who do not show such exaggeration. Thus, a possibility could be that exaggerated amplitude in pantomime—and more generally performance—is associated with theory-of-mind skills and particularly perspective-taking skills. This possibility is partly supported by research on autism spectrum disorders, which has reported an association between pantomime and communication scales.^234^ We say partly because, on the other hand, the difficulties met by patients with schizophrenia do not seem to be explained by their communication disorders and could rather reflect the presence of subtle tool-use disorders strictly speaking.^161^ Regardless, investigating the communicative aspect of pantomime could open new avenues on the interaction between tool use, semantic knowledge and theory of mind.^46^^,^^237^

## Severity versus disconnection

Significant evidence has shown that performance is worse in pantomime than in real tool use. This pattern has been found not only for LBD patients,^15^^,^^54^^,^^60^^,^^71^^,^^73^^,^^109^ but also in Alzheimer’s disease^238^ or in other pathologies such as autism spectrum disorders or schizophrenia (see above), where difficulties are generally found in pantomime but not in real tool use. Two hypotheses have been proposed to account for this pattern^15^^,^^65^: The severity hypothesis versus the disconnection hypothesis. The severity hypothesis is based on the assumption that pantomime is a good proxy for studying real tool-use disorders. Simply, pantomime possesses a better sensitivity to tool-use disorders than real tool use. As discussed so far, this assumption is invalid, because it minimizes the communicative nature of pantomime, which can lead people to produce a demonstration that does not fully correspond to what they can do during real tool use. The disconnection hypothesis posits that pantomime and real tool use are based on distinct cognitive processes. In a way, this hypothesis is close to the communicative hypothesis, according to which pantomime is supported by communicative/language skills and not tool-use skills strictly speaking, and vice versa for real tool use. An important prediction derived from the disconnection hypothesis is that we should also find the opposite pattern, namely, difficulties in real tool use but not in pantomime. This pattern has been reported in two case-studies,^239^^,^^240^ which are subject to methodological flaws.^45^ In other words, evidence rules out the disconnection hypothesis. Instead, it seems that pantomime shares some specific tool-use processes with real tool use, but it also requires additional cognitive processes. This explains why pantomime is more sensitive to diverse pathologies because it is more multidetermined at a cognitive level.

## A neurocognitive model of pantomime

**Figure 10:**
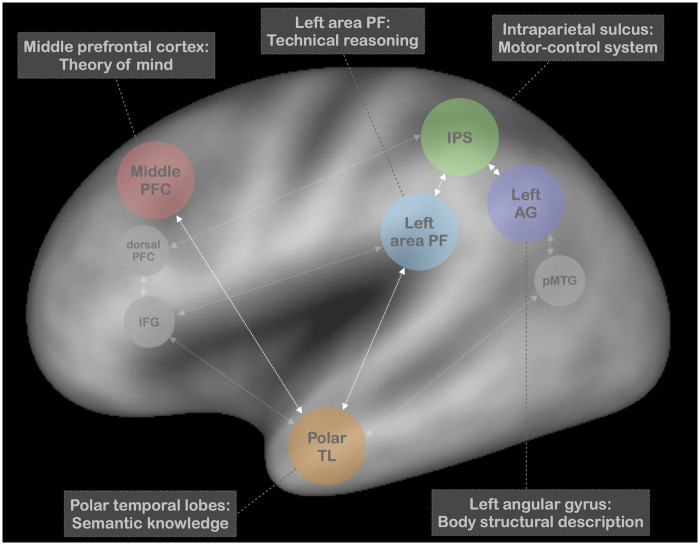
**A neurocognitive model of pantomime of tool use.** This model assumes that technical reasoning (notably the left area PF), the motor-control system (notably the intraparietal sulcus), body structural description (notably the left angular gyrus), semantic knowledge (notably the polar TLs) and potentially theory of mind (notably middle prefrontal cortex) work in concert to produce pantomime. The white arrows indicate the privileged connections between the main brain areas described in the model. Three other brain regions are also represented given their potential involvement in pantomime: Inferior frontal gyrus (IFG), dorsal prefrontal cortex (dorsal PFC) and posterior middle temporal gyrus (pMTG).

This invites us to put forward a new neurocognitive model of pantomime, which is based on the key findings discussed above (Fig.  10; see also Osiurak et al.^131^ and Baumard et al.^210^). Pantomime requires the generation of a mechanical action through technical reasoning (notably the left area PF). The mental simulation of this mechanical action guides the selection of the appropriate motor actions within the motor-control system (notably the IPS). If tools are used towards the body, body structural description is needed (notably the left AG). When the tool is familiar, semantic knowledge (notably the polar TLs) must be recruited to specify the social usages associated with the tool and, thus, to select the appropriate mechanical action to be performed. Finally, theory-of-mind skills might also be involved for perspective taking (notably the middle prefrontal cortex^115^). In this context, pantomime and real tool use might be both impaired after damage to the left area PF because they both crucially rely on technical reasoning. Difficulties in both tasks can also be found after damage to the IPS, because of the obvious requirement of the motor-control system. However, the difficulties met during real tool use do not concern the selection and generation of appropriate mechanical actions but rather the ability to perform appropriate motor actions in order to realize the mechanical action generated (e.g. incorrect grip^144^). Both tasks can also be impaired after damage to the left AG if the real use or demonstration is directed towards the body. Impaired semantic knowledge might have a more deleterious effect in pantomime than in real tool use even if, as discussed above, some subtle difficulties can also be reported in this latter case. Finally, theory-of-mind skills might play a critical role in pantomime because of its communicative nature. These skills can also be recruited during real tool use if the individual is asked to ‘teach’ someone else how to make the tool-use action. In this case, they could be essential to help the individual take the perspective of someone else to facilitate the learning (see above). However, theory-of-mind skills do not intrinsically impact on the individual’s ability to actually use tools with objects.

We acknowledge that the idea that pantomime is cognitively multidetermined is not new.^15^^,^^131^^,^^211^ In the same vein, previous accounts have already proposed that pantomime relies on working memory.^15^^,^^45^^,^^46^^,^^131^ Thus, a working-memory component involving the dorsal prefrontal cortex can be added to our model (Fig.  10). Nevertheless, this implies that difficulties in pantomime should also be reported in patients with dysexecutive syndrome/prefrontal lobe lesions. However, empirical evidence seems to indicate that it is not necessarily the case.^110^ In addition, this also suggests that pantomime could also be impaired in RBD patients. Even if the difficulties are generally greater in LBD patients than in RBD patients, RBD patients performed frequently worse than healthy matched controls.^42^ However, these difficulties can also be explained by other deficits, such as neglect.^241^ In sum, future work is needed to specify whether a specific working-memory component involving the dorsal prefrontal cortex must be added to the model because of the multidetermined nature of pantomime. Note that other interpretations can also be made about the involvement of the dorsal prefrontal cortex in pantomime. For instance, even in relatively simple motor imagery tasks, the dorsal prefrontal cortex along with the IPS has been found to be recruited to form a hierarchical system for flexible, context-adaptive and goal-directed behaviour.^242^ This hierarchical system might be particularly useful for pantomime to help the individual be aware of what they are doing as well as adjust their production to the demands of the context (i.e. self-monitoring).

Two additional brain areas deserve to be mentioned in our neurocognitive model, even if the present findings do not allow us to generate specific hypotheses about their role in pantomime at a cognitive level (Fig.  10). The first is the IFG, which has been repeatedly found to be involved in pantomime.^31^^,^^82^ This brain area has been proposed to play a role in the selection of competing alternatives from semantic memory.^82^ Thus, the left IFG along with the polar TLs might form a ventral network supporting communicative skills,^31^ which is consistent with the presence of poor gestural expression (i.e. gesture accompanying speech) in patients with lesions in anterior temporal and inferior frontal regions.^36^ This interpretation is also supported by other studies combining fMRI and DTI approaches, which have suggested that the left IFG could play a role of explicit cognitive control in testing possible combinations to extract meaning.^106^^,^^242^ The second is the posterior middle temporal gyrus, which has also been found to be involved in pantomime tasks.^17^^,^^19^ This brain area has been proposed to be specialized in the ‘postural component’ of gesture engrams.^17^ This proposal is not supported by the present findings, which have not stressed the specific involvement of the posterior middle temporal gyrus in the posture component of pantomime. Another interpretation is that this brain area (along with AG) contributes to semantic control, which refers to the strategic retrieval of semantic information.^243^^,^^244^ For example, it has been shown that the posterior middle temporal gyrus is preferentially recruited in high semantic control tasks (e.g. associative matching, neighbour judgement) compared to low semantic control tasks (e.g. word-picture matching).^244^ The absence of specific involvement of the left posterior middle temporal gyrus in our different meta-analyses might suggest that the pantomime task does not require high semantic control. Future work is needed to test this possibility.

The neurocognitive model proposed here differs from previous ones in emphasizing the key role of technical reasoning in pantomime. It also aims to identify the brain areas that could be central (hence the ‘notably’) for each cognitive function described (e.g. technical reasoning, body structural description). Importantly, it acknowledges that all the cognitive functions discussed here are necessarily supported by wider brain networks composed of other brain areas, although these latter areas might be less central for the cognitive function concerned. For instance, even if we assume that the left area PF plays a central role in technical reasoning, this kind of reasoning certainly requires other brain areas that might be, nevertheless, not specific to technical reasoning (for discussion about the potential role of the left IFG in technical reasoning, see Reynaud et al.^111^^,^^114^). In this way, our neurocognitive model is in line with previous accounts that have suggested that pantomime might emerge from the interactions between the different brain areas of the ventral, ventro-dorsal and dorso-dorsal streams.^85^^,^^106^^,^^245^

## Apraxia or atechnia?

The literature on apraxia has been dominated for a long time by the gesture-engram hypothesis.^7^^,^^20^^,^^25^^,^^93^^,^^99^^,^^112^^,^^113^^,^^135^^,^^141^^,^^214^ The findings presented here challenge this hypothesis and bring support for the rival technical-reasoning hypothesis (for discussion see Refs.^33^^,^^122–124^^,^^127^^,^^246^^,^^247^). More specifically, the neurocognitive model developed above excludes the existence of gesture engrams and assumes that technical reasoning, the motor-control system, body structural description, semantic knowledge and potentially theory of mind work in concert to produce pantomime. Therefore, this model raises the issue of which clinical manifestations should fall within the scope of apraxia, which owes its existence to the assumption that humans possess a high-level motor component such as gesture engrams. However, if such a component turned out not to exist, then there would be no reason to keep on using the term apraxia for describing pantomime deficits or, more broadly, tool-use disorders. An alternative could be to call these disorders atechnia in reference to technical reasoning, as initially suggested by Gagnepain^129^ (see also Le Gall^130^). However, this proposal is not fully satisfactory because pantomime can be impaired not only after technical-reasoning deficits, which could create confusion. Regardless, in line with the enlightened work of Goldenberg,^3^ the field of apraxia could be more deeply renewed if we adopt the idea that most of the difficulties that we refer to as ‘apraxia’ do not reflect a motor disorder but rather the secondary repercussions of non-motor cognitive disorders on skilled motor performance.^248^

## Supplementary material

Supplementary material is available at *Brain Communications* online.

## Funding

This work was performed within the framework of the LABEX CORTEX (ANR-11-LABX-0042) of Université de Lyon, within the programme ‘Investissements d’Avenir’ (ANR-11- IDEX-0007) operated by the French National Research Agency (ANR).

## Data availability

Data sharing is not applicable to this article as no new data were created or analysed.

## Competing interests 

The authors report no competing interests.

## Supplementary Material

fcab263_Supplementary_Data

## Abbreviations

2 =: Area 2
3b =: Area 3b
4 =: Area 4
6mp =: Area 6mp
6r =: rostral area 6
43 =: Area 43
44 =: Area 44
52 =: Area 52
A1 =: primary auditory cortex
A47r =: area anterior 47r
AG =: angular gyrus
AIP =: anterior intraparietal area
BPT =: body-part-as-tool
GOADI =: goal-directed imitation
IFG =: inferior frontal gyrus
IFJp =: area IFJp
IFSp =: area IFSp
IG =: insular granular complex
IPL =: inferior parietal lobe
IP0 =: intraparietal area 0
IP1 =: intraparietal area 1
IP2 =: intraparietal area 2
IPS =: intraparietal sulcus
LBD =: left brain damage
LIPd =: lateral intraparietal area dorsal
Mbelt =: medial belt complex
MIP =: medial intraparietal area
p32pr =: area p32 prime
Pol1 =: posterior insular area 1
PF =: area PF complex
PFC =: prefrontal cortex
PFcm =: area PFcm
PFm =: area PFm complex
PFop =: area PF opercular
PFt =: area PFt
PGi =: area PGi
PALS-B12 =: population average landmark- and surface-based atlas
PEF =: premotor eye field
POS2 =: parieto-occipital sulcus area 2
pMTG =: posterior middle temporal gyrus
OP4 =: area OP4/PV
RBD =: right brain damage
RI =: retroinsular cortex
TL =: temporal lobe
V2 =: second visual area
V7 =: seventh visual area
VIP =: ventral intraparietal complex
