# Supplementary material for: Pantomime of tool use: looking beyond apraxia
Source: Brain Commun. 2021 Oct 30;3(4):fcab263. doi: 10.1093/braincomms/fcab263 (PMC8936430; doi:10.1093/braincomms/fcab263)
Supplement: fcab263_Supplementary_Data [file fcab263_Supplementary_Data.pdf]

Pantomime of tool use: Looking beyond apraxia

François Osiurak[1,2], Emanuelle Reynaud[1], Josselin Baumard[3],
Yves Rossetti[4,5], Angela Bartolo[2,6], & Mathieu Lesourd[7,8]

[1]Laboratoire d'Etude des Mécanismes Cognitifs (EA3082), Université de Lyon, France
[2]Institut Universitaire de France, Paris, France
[3]Normandie Univ., UNIROUEN, CRFDP (EA7475), Rouen, France
[4]Centre de Recherche en Neurosciences de Lyon, Trajectoires Team, CNRS U5292, Inserm U1028, Université de Lyon, France
[5]Mouvement, Handicap, et Neuro-Immersion, Hospices Civils de Lyon et Centre de Recherche en Neurosciences de Lyon, Hôpital Henry Gabrielle, St Genis Laval, France
[6]Univ. Lille, CNRS, UMR9193, SCALab – Sciences Cognitives et Sciences Affectives, Lille, France
[7]Laboratoire de Recherches Intégratives en Neurosciences et Psychologie Cognitive (UR481), Université de Bourgogne Franche-Comté, Besançon, France
[8]MSHE Ledoux, CNRS, Université de Bourgogne Franche-Comté, Besançon, France

Correspondence

François Osiurak, Laboratoire d'Etude des Mécanismes Cognitifs (EA 3082), Institut de Psychologie, 5, avenue Pierre Mendès-France, 69676 Bron Cedex, France.
Email: Francois.Osiurak@univ-lyon2.fr

Supplementary Methods

Brain lesion studies

For the selection of brain lesions studies, we used the terms 'apraxia' AND 'brain lesions' to identify studies of interest for each of our analyses (i.e., real tool use, pantomime of tool use, and imitation of meaningless gestures). We used the search engine "PubMed". This search returned 773 results at the date of 22/12/2020. We restricted our selection for each analysis to studies that met a series of selection criteria:

1. Only patients presenting exclusively left brain-damaged patients were considered.
2. Single case studies were not included.
3. Only studies using lesion mapping method were included (e.g., lesion-subtraction analysis or voxel-based lesion symptom mapping).
4. Reports had to provide lesion peaks data or at least overlay lesion plots associated with selective disturbances.

When coordinates lesions were available, we depicted the location of the reported lesion sites on flat-map representations of a left hemisphere (PALS-B12; Population-Average, Surface and Landmark-based human cortical atlas[1]). If only lesion plots were reported in the study, we made several transformations before depicting the lesion sites on the flatmap representation, by following a three-step method. This three-step method is completely described in Lesourd et al.[2] (see also[3]). Finally, we used the parcellation of Glasser et al.[4] to identify the brain areas of interest and considered a brain area as a brain area of interest when a brain area was reported in all the "modalities" (e.g., modality for pantomime of tool use or type of posture for imitation of meaningless postures) or in at least 50% of the conditions included for a given "modality" (e.g., visual modality for pantomime of tool use).

Neuroimaging studies

For the selection of neuroimaging studies, we used the terms 'pantomime' AND 'neuroimaging' to identify studies of interest for our analysis on pantomime of tool use, and 'imitation' AND 'neuroimaging' to identify studies of interest for our analysis on imitation of meaningless gestures. We used the search engine "PubMed". This search

returned 90 results at the date of 22/12/2020 for 'pantomime' AND 'neuroimaging' and 557 results at the date of 22/12/2020 for 'imitation' AND 'neuroimaging'. We also conducted an additional search with the terms 'gesture' AND 'neuroimaging', which returned 448 results at the date of 22/12/2020. We restricted our selection for each analysis to studies that met a series of selection criteria:

1. Reviews were excluded.
2. Studies had to use functional magnetic resonance imaging (fMRI) or positron emission tomography (PET) as imaging modality.
3. Only neurologically healthy adults were included.
4. Neuroimaging results had to be based on whole-brain scanning. Regions of interest analyses were therefore excluded from our selection.
5. The complete list of activation peaks (i.e., foci) of main effects with their coordinates had to be reported in a stereotactic space.

The meta-analysis of neuroimaging studies was conducted using the revised version[5,6] of the Activation Likelihood Estimation[7] (ALE) method, as implemented by the GingerALE 2.3 software (http://www.brainmap.org/ale/). The methods employed here is the same as the one described by Reynaud et al.[8] (see also[2,9,10]). Finally, the resulting thresholded ALE maps were visualized on flatmap representations of a standardized brain atlas (PALS-B12) using Caret, version 5.65[1] (http://brainmap.wustl.edu/caret.html).

Studies of patients with selective semantic deficits

For the selection of studies of patients with selective semantic deficits, we did not use the search engine "PubMed" because of the difficulty to use the appropriate terms. Indeed, in such studies, the assessment of apraxia can be very "peripheral" and difficult to be identified by a search engine. Instead, we conducted a bibliographical search from the key papers on the topic[11–13]. We reported only the papers in which the data on semantic knowledge tasks and pantomime/single tool use were available for each patient. Note that we did not find any paper in which a correlation coefficient was mentioned between semantic knowledge tasks and pantomime/single tool use without the individual data for each patient. Said differently, the data reported in the present review correspond to all the data we have obtained on this topic.

References

1. Van Essen DC, Drury HA, Dickson J, Harwell J, Hanlon D, Anderson CH. An integrated software suite for surface-based analyses of cerebral cortex. *J Am Med Informatics Assoc*. 2001;8(5):443-459.
2. Lesourd M, Osiurak F, Baumard J, Bartolo A, Vanbellingen T, Reynaud E. Cerebral correlates of imitation of intransitive gestures: An integrative review of neuroimaging data and brain lesion studies. *Neurosci Biobehav Rev*. 2018;95:44-60.
3. Niessen E, Fink GR, Weiss PH. Apraxia, pantomime and the parietal cortex. *NeuroImage Clin*. 2014;5:42-52.
4. Glasser MF, Coalson TS, Robinson EC, et al. A multi-modal parcellation of human cerebral cortex. *Nature*. 2016;536(7615):171-178.
5. Eickhoff SB, Laird AR, Grefkes C, Wang LE, Zilles K, Fox PT. Coordinate-based activation likelihood estimation meta-analysis of neuroimaging data: A random-effects approach based on empirical estimates of spatial uncertainty. *Hum Brain Mapp*. 2009;30(9):2907-2926.
6. Eickhoff SB, Bzdok D, Laird AR, Kurth F, Fox PT. Activation likelihood estimation meta-analysis revisited. *Neuroimage*. 2012;59(3):2349-2361.
7. Turkeltaub PE, Eden GF, Jones KM, Zeffiro TA. Meta-analysis of the functional neuroanatomy of single-word reading: method and validation. *Neuroimage*. 2002;16(3):765-780.
8. Reynaud E, Lesourd M, Navarro J, Osiurak F. On the neurocognitive origins of human tool use: A critical review of neuroimaging data. *Neurosci Biobehav Rev*. 2016;64:421-437.
9. Faye A, Jacquin-Courtois S, Reynaud E, Lesourd M, Besnard J, Osiurak F. Numerical cognition: A meta-analysis of neuroimaging, transcranial magnetic stimulation and brain-damaged patients studies. *NeuroImage Clin*. 2019;24.
10. Reynaud E, Navarro J, Lesourd M, Osiurak F. To watch is to work: A review of neuroimaging data on tool use observation network. *Neuropsychol Rev*. 2019;29(4):484-497.
11. Hodges JR, Spatt J, Patterson K. “What” and “how”: Evidence for the dissociation of object knowledge and mechanical problem-solving skills in the human brain. *Proc Natl Acad Sci U S A*. 1999;96(16):9444-9448.
12. Hodges JR, Bozeat S, Lambon Ralph MA, Patterson K, Spatt J. The role of conceptual knowledge in object use: Evidence from semantic dementia. *Brain*. 2000;123(9):1913-1925.
13. Bozeat S, Lambon Ralph MA, Patterson K, Hodges JR. When objects lose their meaning: What happens to their use? *Cogn Affect Behav Neurosci*. 2002;2(3):236-251.

Supplementary Information

All the references mentioned in the tables are cited in the main text.

Supplementary Table 1. Summary of the brain lesion studies included in "Real tool use"

Study	*N*	Task	Coordinates	Table/figure
Goldenberg & Spatt (2009)	38	Familiar tool use	Lesion plots	Fig. 1
Goldenberg & Spatt (2009)	38	Mech. problem solving	Lesion plots	Fig. 1
Randerath et al. (2010)	42	Familiar tool use	Available	Table 1 (Erroneous tool use)
Mengotti et al. (2013)	57	Familiar tool use	Available	Table 5 (Ideational apraxia)
Martin, Beume, et al. (2016)	136	Familiar tool use	Lesion plots	Fig. 3A
Salazar-Lopez et al. (2016)	31	Familiar tool use	Lesion plots	Fig. 5A

N, Number of left brain-damaged patients included in the study. Mech. problem solving, Mechanical problem solving.

Supplementary Table 2. Summary of the brain lesion studies included in "Pantomime of tool use"

Study	*N*	Modality	Coordinates	Table/figure
Hermsdörfer et al. (2013)	23	Visual	Lesion plots	Fig. 6 (Panto)
Kalénine et al. (2013)	23	Visual	Lesion plots	Fig. 6A
Buxbaum et al. (2014)	71	Visual	Available	Table 2 (GestTool)
Hoeren et al. (2014)	96	Visual	Lesion plots	Fig. 4A
Tarhan et al. (2015)	131	Visual	Available	Table 2 (AP)
Watson & Buxbaum (2015)	31	Visual	Lesion plots	Fig. 5A*
Martin, Nitschke, et al. (2016)	36	Visual	Lesion plots	Fig. 8C (PantoTool)
Weiss et al. (2016)	50	Visual	Lesion plots	Fig. 4A
Dressing et al. (2019)	48	Visual	Lesion plots	Fig. S2C (Pantomime)
Tessari et al. (2007)	22	Imitation	Lesion plots	Fig. 4B
Mengotti et al. (2013)	57	Imitation	Available	Table 5 (Meaningful)°
Buxbaum et al. (2014)	71	Imitation	Available	Table 2 (ImTool)
Martin, Nitschke, et al. (2016)	36	Imitation	Lesion plots	Fig. 8C (ImiTool)
Dressing et al. (2018)	156	Imitation	Lesion plots	Fig. S1A
Dressing et al. (2019)	48	Imitation	Lesion plots	Fig. S2C (Imitation)
Goldenberg et al. (2007)	44	Verbal[#]	Lesion plots	Fig. 1B
Manuel et al. (2013)	150	Verbal	Lesion plots	Fig. 1B
Goldenberg & Randerath (2015)	96	Verbal[#]	Lesion plots	Fig. 2 (Pantomime)
Finkel et al. (2018)	67	Verbal[#]	Lesion plots	Fig. 2A

N, Number of left brain-damaged patients included in the study. *, This study reports a significant cluster of 81981 voxels in the internal capsule (white matter) in Table 3 (MNI coordinates: x = -24, y = -24, z =12). Given the size of the cluster, it appeared inappropriate to report only one coordinate. This explains why we used the three-step method based on the lesion plots depicted in the Figure 5 of this study. **, The score reported here corresponds to a composite score "pantomime + symbolic gestures". [#], In these three studies, a picture of the object is shown along with the verbal command. We considered that the verbal modality is privileged over the visual modality and assigned these three studies to the "verbal" category.

Supplementary Table 3. Summary of the neuroimaging studies included in "Pantomime of tool use"

**Study**	***N***	**Method**	**Modality**	**Table**
Choi et al. (2001)	10	fMRI	Verbal	Table 2 (Right hand)
Ohgami et al. (2004)	18	fMRI	Verbal	Table 1 (Mime with right hand)
Johnson-Frey et al. (2005)	13	fMRI	Verbal	Table 1
Fridman et al. (2006)	19	fMRI	Verbal	Table 1a
Montgomery et al. (2007)	14	fMRI	Verbal	Table 1 (Value for do)
Bohlhalter et al. (2009)	15	fMRI	Verbal	Table 1 (Transitive/Right)
Króliczak & Frey (2009)	12	fMRI	Verbal	Table SA
Emmorey et al. (2011)	14	PET	Verbal	Table 2 (Pantomime)
Mäki-Marttunen et al. (2014)	20	fMRI	Verbal	Table 1a
Mäki-Marttunen et al. (2014)	20	fMRI	Verbal	Table 1b
Hermsdörfer et al. (2007)	23	fMRI	Visual	Table 1 (Event 2)
Higuchi et al. (2007)	8	fMRI	Visual	Table 2 (Imagery)
Imazu et al. (2007)	12	fMRI	Visual	Table 2 (Pantomime/Tool)
Imazu et al. (2007)	12	fMRI	Visual	Table 2 (Imagining/Tool)
Vingerhoets et al. (2009)	15	fMRI	Visual	Table 2 (Use … >move neutral shapes)
Peran et al. (2010)	12	fMRI	Visual	Table 2 (MSoA>GenA)
Vingerhoets et al. (2011)	16	fMRI	Visual	Table 1 (Planning/Familiar tools)
Wadsworth et al. (2011)	32	fMRI	Visual	Table 2 (Imagined tool use…)
Vingerhoets et al. (2013)	10	fMRI	Visual	Table 3
Lausberg et al. (2015)	15	fMRI	Visual	Table 1 (Conjunction P lh…)
Vry et al. (2015)	24	fMRI	Visual	Table 2 (Pantomime)

Supplementary Table 4. Summary of the brain lesion studies included in "Kinematics and Posture"

**Study**	***N***	**Component**	**Coordinates**	**Table/figure**
Manuel et al. (2013)	150	Kinematics	Lesion plots	Fig. S1C
Buxbaum et al. (2014)	71	Kinematics	Available	Table 3 (ImTool kinematics)
Hoeren et al. (2014)	96	Kinematics	Lesion plots	Fig. S1A*
Dressing et al. (2018)	156	Kinematics	Lesion plots	Fig. 5A
Finkel et al. (2018)	67	Kinematics	Lesion plots	Fig. 2C
Manuel et al. (2013)	150	Posture (BPT)	Lesion plots	Fig. S1D
Finkel et al. (2018)	67	Posture (BPT)	Lesion plots	Fig. 2D
Dressing et al. (2018)	156	Posture (Grip)	Lesion plots	Fig. 5B
Finkel et al. (2018)	67	Posture (Grip)	Lesion plots	Fig. 2B
Buxbaum et al. (2014)	71	Posture (BPT/Grip)	Available	Table 3 (GestTool posture)
Buxbaum et al. (2014)	71	Posture (BPT/Grip)	Available	Table 3 (ImTool posture)
Watson & Buxbaum (2015)	31	Posture (BPT Grip)	Available	Table 3 (Conjunction)

N, Number of left brain-damaged patients included in the study. *, non-recognizable errors.

Supplementary Table 5. Summary of the brain lesion studies included in "Imitation of meaningless postures"

**Study**	***N***	**Posture**	**Coordinates**	**Table/figure**
Mengotti et al. (2013)	57	Hand/finger	Available	Table 6 (Meaningless)
Hoeren et al. (2014)	96	Hand/finger	Lesion plots	Fig. 2
Martin, Beume, et al. (2016)	136	Hand/finger	Lesion plots	Fig. 8B
Weiss et al. (2016)	50	Hand/finger	Lesion plots	Fig. 4C
Pizzamiglio et al. (2019)	387	Hand/finger	Available	Table 4 (Gesture imitation)
Goldenberg & Karnath (2006)	44	Hand	Lesion plots	Fig. 3A
Buxbaum et al. (2014)	71	Hand	Available	Table 2 (ImNov)
Goldenberg & Randerath (2015)	96	Hand	Lesion plots	Fig. 2 (Hand)
Achilles et al. (2019)	293	Hand	Lesion plots	Fig. S1
Tessari et al. (2021)	36	Hand	Lesion plots	Fig. 4C (Blue)
Goldenberg & Karnath (2006)	44	Finger	Lesion plots	Fig. 3B
Goldenberg & Randerath (2015)	96	Finger	Lesion plots	Fig. 2 (Finger)
Achilles et al. (2019)	293	Finger	Lesion plots	Fig. 3A
Tessari et al. (2021)	36	Finger	Lesion plots	Fig. 4C (Red)
Dafsari et al. (2019)	*27*	*Body struct. desc.*	*Lesion plots*	*Fig. 4C*

N, Number of left brain-damaged patients included in the study. Body struct. desc., body structural description task.
*, non-recognizable errors.

Supplementary Table 6. Summary of the neuroimaging studies included in "Imitation of meaningless postures"

**Study**	***N***	**Method**	**Posture**	**Table**
Tanaka & Inui (2002)	12	fMRI	Finger	Table 1 (Finger)
Tanaka & Inui (2002)	12	fMRI	Hand	Table 1 (Hand)
Koski et al. (2003)	8	fMRI	Finger	Tables 1 & 2 (…vs. control motor task)
Koski et al. (2003)	8	fMRI	Finger	Table 3
Buccino et al. (2004)	12	fMRI	Finger	Table 1
Chaminade et al. (2005)	12	fMRI	Hand	Table 4 (Imitating)
Mulhau et al. (2005)	12	fMRI	Hand/Finger	Table 2
Rumiati et al. (2005)	10	PET	Hand	Table 3 (Main effect of ML movements)
Jackson et al. (2005)	16	fMRI	Hand	Table 1
Suchan et al. (2008)	12	PET	Finger	Table 2
Adamovich et al. (2009)	13	fMRI	Finger	Table 1
Watanabe et al. (2011)	15	fMRI	Finger	Table 2 (First person/anatomical)
Watanabe et al. (2011)	15	fMRI	Finger	Table 2 (First person/specular)
Watanabe et al. (2011)	15	fMRI	Finger	Table 2 (Third person/anatomical)
Watanabe et al. (2011)	15	fMRI	Finger	Table 2 (Third person/specular)
Kruger et al. (2014)	20	fMRI	Hand	Table 1
Vingerhoets et al. (2015)	17	fMRI	Finger	Table 2 (ImNov>PanTool)
Watanabe et al. (2017)	17	fMRI	Finger	Table 2
Watanabe et al. (2017)	17	fMRI	Finger	Table 3

Supplementary Table 7. Summary of the studies of patients with selective semantic deficits

Study	*N*	Task	Description
Coccia et al. (2004)	8*	Pantomime	*Semantic knowledge:* Visual naming (Table 3).<br>*Pantomime:* Visual pantomime and Pantomime-to-name (Table 3).
Nishio et al. (2006)	6	Pantomime	*Semantic knowledge:* Picture naming (Table 1).<br>*Pantomime:* Action imitation test/Instrumental (Table 2).
Silveri & Ciccarelli (2009)	5	Pantomime	*Semantic knowledge:* Object naming (Table 5).<br>*Pantomime:* Pantomime execution (Table 5).
Lesourd et al. (2017)	16	Pantomime	*Semantic knowledge:* Functional matching task (Personal data).<br>*Pantomime:* Pantomime of tool use (Personal data).
Hodges et al. (2000)	9	Single tool use	*Semantic knowledge:* Visual associative knowledge (Fig.3; Composite score: Recipient, location, purpose).<br>*Single tool use:* Object use (Fig. 4; Composite score: Hold, orientation, movement).
Bozeat et al. (2002)	8	Single tool use	*Semantic knowledge:* Visual associative knowledge (Fig.2; Composite score: Recipient, function, action).<br>*Single tool use:* Single object use (Fig. 7).
Silveri & Ciccarelli (2009)	5	Single tool use	*Semantic knowledge:* Object naming (Table 5).<br>*Single tool use:* Object use (Table 5).
Nelissen et al. (2010)	9	Single tool use	*Semantic knowledge:* Associative-semantic task (Table 1).<br>*Single tool use:* Object use (Table 1).
Baumard et al. (2016)	16	Single tool use	*Semantic knowledge:* Functional matching task (Personal data).<br>*Single tool use:* Single tool use (Personal data).

*, two patients who were assessed over a four-year longitudinal period.
